# A Stage-Aware Cascaded Detection–Segmentation Framework for Leaf Phenotyping and Leaf Dry Biomass Estimation of Pepper Seedlings

**DOI:** 10.3390/plants15121912

**Published:** 2026-06-20

**Authors:** Han Li, Dongyuan Shi, Hui Shi, Ming Li, Ming Diao

**Affiliations:** 1 College of Agriculture, Shihezi University, Shihezi 832003, China; lihan20321206@gmail.com (H.L.); ayeenchante@gmail.com (D.S.); 2 Research Center of Information Technology, Beijing Academy of Agriculture and Forestry Sciences, Beijing 100097, China; huishi180@gmail.com; 3 Department of Informatics, University of Almería, CeiA3, CIESOL, 04120 Almería, Spain

**Keywords:** pepper seedlings, deep learning, semantic segmentation, phenotypic correction, leaf dry biomass estimation

## Abstract

Quantitative phenotyping of pepper seedlings is important for greenhouse plug tray seedling cultivation, but it remains constrained by inefficient manual monitoring, complex greenhouse backgrounds, and growth-stage-dependent discrepancies between two-dimensional image traits and actual leaf biomass. In this study, a cascaded vision framework with stage-specific morphological correction was developed for nondestructive seedling phenotyping. The framework integrated Visual Dynamic Momentum YOLO (VDM-YOLO) for individual seedling localization and growth-stage recognition, Variance Guided Strip Ghost Gated UNet (VSG-UNet) for lightweight, high-resolution leaf segmentation, and a stage-aware correction model for leaf dry biomass estimation. In performance evaluation, VDM-YOLO achieved a mean average precision at an intersection over union threshold of 0.5 (mAP_0.5_) of 89.27%, improving mAP_0.5_ by 1.82 percentage points over YOLOv12. VSG-UNet achieved a mean intersection over union (mIoU) of 83.9% and a Dice coefficient of 81.8%, while reducing floating point operations (FLOPs) and parameters by 44.2% and 61.2%, respectively, compared with U-Net. After stage-aware calibration, the coefficient of determination (*R*^2^) between segmented area and leaf dry weight increased from 0.764 to 0.813, and the root mean square error (RMSE) decreased from 0.0210 g to 0.0190 g. These results demonstrated that the proposed framework provided a proof of concept approach based on RGB images for the nondestructive assessment of leaf area and leaf dry biomass in pepper seedlings under restricted experimental conditions.

## 1. Introduction

Pepper [*Capsicum annuum* (L.)] is an economically important crop worldwide, and seedling growth strongly influences later flowering, fruit set, yield, and fruit quality [1]. With the development of greenhouse plug tray seedling cultivation, seedling management increasingly requires digital and precise monitoring. Compared with several other vegetable seedlings, pepper seedlings present specific challenges for image-based phenotyping. At the early true leaf stages, pepper leaves are relatively small and often show variable inclination, while morphological differences among the two-, four-, and six-true-leaf stages are subtle. In addition, leaf overlap, plug tray cells, substrate particles, wet regions, and shadows introduce strong background interference in greenhouse images. These characteristics make both growth-stage discrimination and leaf boundary extraction difficult when using standard RGB images. However, accurate assessment during the two- to six-true-leaf stages is particularly important because these stages are critical for evaluating seedling vigor, selecting high quality seedlings, and guiding water and fertilizer management. Traditional manual measurements remain time consuming, subjective, and often destructive [2,3]. Although deep learning has advanced object detection and semantic segmentation in crop phenotyping, reliable phenotypic extraction under complex greenhouse conditions remains challenging.

The You Only Look Once (YOLO) [4] family of algorithms has been widely used in agricultural vision tasks, including seedling counting, pest and disease monitoring, crop identification, and classification of developmental stages, owing to its fast inference, simple architecture, and end-to-end training capability [5]. To improve green pepper detection under complex environmental conditions, Pepper-YOLO was developed to enhance detection and localization accuracy while reducing the number of model parameters by 38.3% [6]. YOLO-Chili was designed for pepper detection in complex scenes, improving detection accuracy and real time performance by integrating multi scale feature fusion, attention mechanisms, and lightweight pruning [7]. Beyond object localization, YOLO has also been applied to identifying the stage of growth of crops. For hydroponic chili plants, YOLOv8 was used to identify vegetative, flowering, and fruiting stages, with dual view training data improving model generalization and YOLOv8m achieving an mAP_0.5_ of 92.63% [8]. To address small object size and occlusion at the seedling stage, a lightweight YOLOv4-based model using MobileNetV3-Small and an attention mechanism achieved high-precision, low-latency weed detection in carrot seedling fields [9].

Compared with object detection, semantic segmentation provides pixel-level leaf delineation, better supporting leaf area estimation and biomass-related analysis. For example, EffUNet++ used an EfficientNet-B4 encoder–decoder framework to improve multi-level leaf feature fusion while reducing computational complexity [10]. For citrus leaf disease monitoring, a dual-path ViT-U-Net improved complex disease texture segmentation by combining convolutional feature extraction with Transformer-based global modeling [11]. Mobile vision has also been used for fruit tree structural segmentation, where a VGG16-based U-Net achieved a mean IoU of 93.33% for segmenting trunks, primary branches, and young branches in cherry trees [12]. To enhance segmentation robustness under greenhouse imaging conditions, ImageNet-pretrained U-Net variants were evaluated for tomato leaf and fruit segmentation using images acquired under uncontrolled lighting [13]. Although semantic segmentation has been widely used for crop organ extraction and phenotypic analysis, maintaining segmentation accuracy and boundary detail while preserving model efficiency remains a major challenge for seedling segmentation in complex backgrounds. In seedling phenotyping at high throughput, methods based on U-Net have been used for hypocotyl length measurement from low-quality scanner or smartphone images and for hypocotyl root segmentation in wild type *Col-0* and *aux17* mutant *Arabidopsis* seedlings [14,15]. To improve seedling contour extraction under uneven illumination, an automatic contour determination and data augmentation method was proposed, offering a useful strategy for seedling contour segmentation in complex backgrounds [16]. Nevertheless, direct full-image segmentation of high-resolution panoramic seedling images can still introduce substantial redundant background information and high computational cost, making it difficult to balance segmentation accuracy and efficiency.

For nondestructive seedling quality evaluation, projected canopy area and vegetation indices have been used to predict the growth and quality of basil seedlings, indicating that morphometric and color-related traits can provide complementary information for seedling assessment [17]. In cucumber seedlings, leaf area and greenness were further estimated at both the leaf and whole seedling scales, demonstrating the value of combining area-based and greenness-related traits for seedling phenotyping [18]. These studies highlight the value of integrating morphometric and color-related traits for nondestructive seedling evaluation. However, accurate quantitative phenotyping in plug tray nursery images still requires reliable individual seedling localization, growth-stage discrimination, fine-scale leaf segmentation, and correction of stage-dependent bias between projected image traits and biomass. These requirements are particularly important for pepper seedlings, whose early stage leaves are small, inclined, and easily affected by background interference and overlap.

To address the need for accessible and user friendly phenotyping methods, this study develops a quantitative analysis framework for pepper seedlings using standard RGB images and conventional imaging equipment, without relying on specialized three-dimensional, RGB-D, or hyperspectral imaging systems. The novelty of this study lies mainly in the construction of a cascaded detection segmentation and correction framework for pepper seedling phenotyping. In this framework, an improved YOLO-based model was used to localize individual seedlings and identify growth stages, providing cropped Region of Interest (ROI) images and stage information for downstream analysis. A lightweight improved U-Net model was then developed to segment leaf regions within the localized ROI, thereby reducing background interference from plug trays, substrate, and neighboring seedlings. Finally, growth-stage information was incorporated to correct segmented leaf area and improve its relationship with leaf dry biomass. The main contributions of this study are as follows:

1. A cascaded quantitative phenotyping framework was established by integrating seedling localization, growth-stage recognition, ROI-based leaf segmentation, segmented area correction, and leaf dry biomass estimation.
2. An improved YOLO-based detection model was developed to enhance individual seedling localization and growth-stage recognition under complex plug tray greenhouse backgrounds.
3. A lightweight improved U-Net segmentation model was developed to improve fine-scale leaf segmentation within cropped seedling ROIs while reducing model complexity.
4. A growth-stage-aware area correction strategy was established to reduce the bias between segmented projected leaf area and leaf dry biomass, providing a basis for nondestructive leaf dry biomass estimation in pepper seedlings.

These models enable nondestructive estimation of leaf area and leaf dry biomass from RGB images, providing methodological support for seedling status assessment and high-quality seedling selection.

## 2. Materials and Methods

### 2.1. Image Acquisition and Dataset Creation

**Figure 1:**
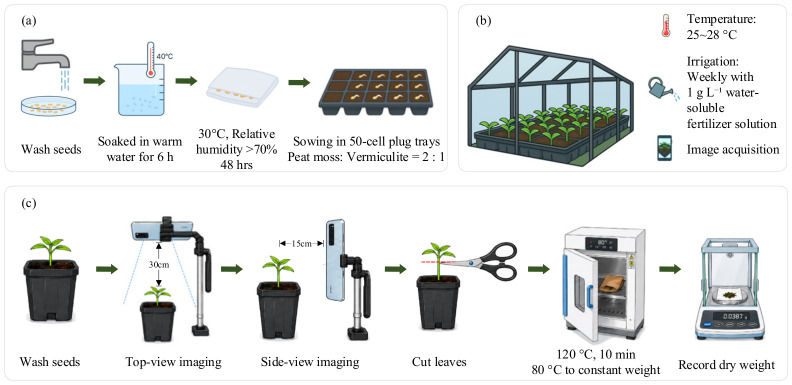
Workflow of seedling preparation, cultivation, image acquisition, and biomass measurement: (**a**) seed preparation and sowing; (**b**) greenhouse cultivation; (**c**) schematic workflow of individual seedling image acquisition, sample drying, and leaf dry weight determination.

The experiment was conducted in December 2024 in a greenhouse at the College of Agriculture, Shihezi University, China (44.3191° N, 86.0672° E). The plant material consisted of the “Siping Tou” sweet pepper cultivar (Xinjiang Jienong Seed Co., Ltd., Changji, Xinjiang, China). “Siping Tou” is a local pepper cultivar in Xinjiang. Its mature fruits are usually square bell shaped, with large fruit size, thick flesh, and relatively strong adaptability. Compared with long fruit and line pepper cultivars, “Siping Tou” seedlings generally show a relatively compact plant type, broader leaves, and stronger stems, providing suitable material for the initial development of a pepper seedling phenotyping framework. Seeds were soaked, pregerminated, and sown in 50-cell plug trays containing a peat moss and vermiculite mixture at a 2:1 volume ratio (Figure 1a). The greenhouse was maintained at 25–28 °C, and the relative air humidity was maintained at 65–70%. Because the seedlings received limited natural light, LED plant growth lamps were used as the main supplemental light source. The LED lighting was provided from 08:00 to 21:00 each day. Each cultivation layer was equipped with four T8 full spectrum white LED plant growth lamps, each 1200 mm in length and 18 W in power. The distance between the LED lamps and the seedling canopy was approximately 40–45 cm. Irrigation was performed every two days, with approximately 2000 mL of water applied each time. Seedlings received weekly irrigation with a 1 g L^−1^ water soluble fertilizer solution (Figure 1b). The cultivation period of pepper seedlings lasted 45 days and included three growth stages: the two-, four-, and six-true-leaf stages.

Seedling images were captured from top down, lateral, and oblique perspectives using a Huawei P40 Pro smartphone (Huawei Technologies Co., Ltd., Shenzhen, China) at different times of the day under natural illumination. Background elements, including tray edges and exposed growing medium, were retained to reflect realistic greenhouse conditions. For standardized individual plant sampling, seedlings showing normal growth at each stage were first screened. Seedlings with visible disease symptoms, mechanical damage, severe growth retardation, or abnormal morphology were excluded. From the remaining normally growing seedlings, 37 plants were randomly selected from each growth stage, yielding a total of 111 seedlings. After the seedlings had been selected from the corresponding growth stages, they were transplanted into individual pots and imaged immediately for standardized single seedling phenotypic measurement. Images were acquired from fixed positions with a millimeter scale ruler in the field of view for leaf area calibration. Top view images were captured with a 30 cm vertical distance to the canopy, while side view images were captured with a 15 cm horizontal distance from the plant center to extract leaf inclination information. The true leaves were cut and oven dried immediately after imaging for leaf dry weight determination. This procedure minimized possible morphological changes caused by transplanting. The individual seedling images were processed using the trained detection and segmentation models to obtain segmented leaf area, which was then used for subsequent area correction, leaf dry weight fitting, and independent validation.

Following imaging, all true leaves of each seedling were carefully cut and placed in paper envelopes. The samples were heat-treated at 120 °C for 10 min to rapidly stop physiological activity and moisture exchange, and then oven-dried at 80 °C in a WGL-230D drying oven (Taisite, Tianjin, China) until constant weight. Leaf dry weight was determined using an FA2004 electronic analytical balance (Puchun, Shanghai, China), following the general principle of plant dry mass measurement based on oven drying to constant mass [19] (Figure 1c). Cotyledons, stems, and roots were not included because the image segmentation model was designed to extract true leaf regions, and the subsequent area correction and estimation were based on leaf area traits. Therefore, dry weight in this study refers to true leaf dry weight rather than whole seedling dry weight. Leaf dry weight data were subsequently recorded. Among these samples, 96 seedlings were used for phenotypic segmentation calibration, while 15 seedlings served as an independent validation set for leaf dry biomass estimation.

**Figure 2:**
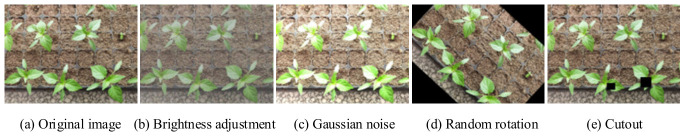
Representative examples of data augmentation methods: (**a**) original image; (**b**) brightness adjustment; (**c**) Gaussian noise; (**d**) random rotation; (**e**) cutout.

The image dataset was partitioned using stratified random sampling. For the object detection task, the dataset was divided into training, testing, and validation sets at a ratio of 7:2:1. After augmentation, the training set was expanded to 3582 images, resulting in a total of 3836 images. For the semantic segmentation task, the dataset was divided into training and validation sets at a ratio of 8:2. The training set was augmented to 1876 images, leading to a total of 1993 images. To avoid data leakage, dataset partitioning was performed before data augmentation. Augmentation was applied only to the training subset, and augmented versions of the same original image were not assigned to the validation or test subsets. The augmentation strategies aimed at improving model generalization and reducing the risk of overfitting, and mainly included brightness adjustment, Gaussian noise, random rotation, and Cutout. Specifically, brightness adjustment included both darkening and brightening operations; the darkening factor was randomly selected from 0.35–0.65, whereas brightening was performed with an alpha value of 1.45–2.10 and a beta value of 10–40. Gaussian noise was added with a mean of 0 and a standard deviation randomly selected from 28–55. Random rotation was performed within 25–60° in either the clockwise or counterclockwise direction. Cutout was implemented as black block occlusion using one to two square masks, with the side length of each mask set to 12–30% of the shorter image side. Representative augmented samples are shown in Figure 2, and the detailed composition of the dataset is presented in Table 1.

### 2.2. VDM-YOLO Detection Network

**Figure 3:**
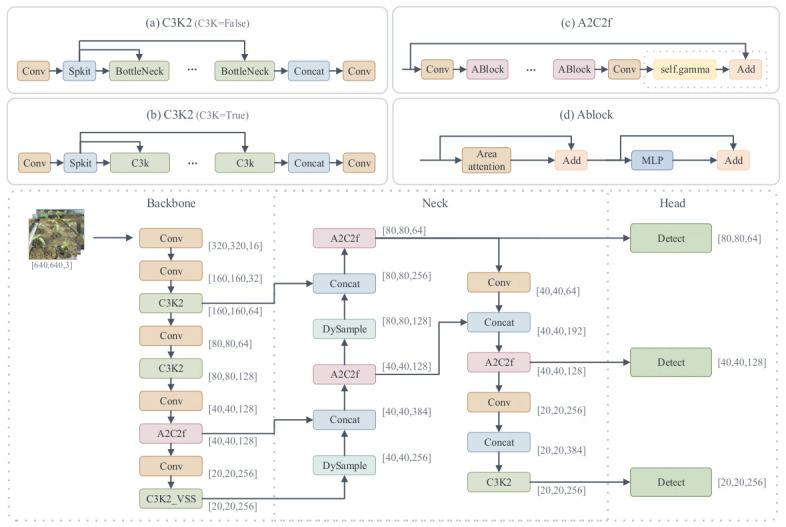
The structures of VDM-YOLO: (**a**) C3K2 module with C3K = False; (**b**) C3K2 module with C3K = True; (**c**) A2C2f module; (**d**) Ablock module.

Growth-stage classification and seedling localization aim to extract the ROI of each seedling from complex images. YOLOv12 was selected as the baseline model and improved in three aspects: feature extraction, upsampling reconstruction, and loss optimization. Figure 3 shows the overall architecture of the resulting VDM-YOLO model, which was designed to enhance seedling localization and stage of growth classification under complex background conditions and to provide accurate inputs for subsequent leaf segmentation.

#### 2.2.1. Visual State Space C3k2 Module

Under complex greenhouse conditions, seedling recognition requires both local texture extraction and global context modeling. Conventional convolutional networks are effective for local feature extraction but have limited ability to model long range dependencies, making them susceptible to background interference. Samba [20] introduced visual state space modeling and achieved a favorable balance between global modeling capability and computational efficiency. Inspired by this design, a visual state space mechanism was integrated into the C3k2 module to construct the Visual State Space C3k2 (C3k2_VSS) module, thereby enhancing contextual feature representation.

**Figure 4:**
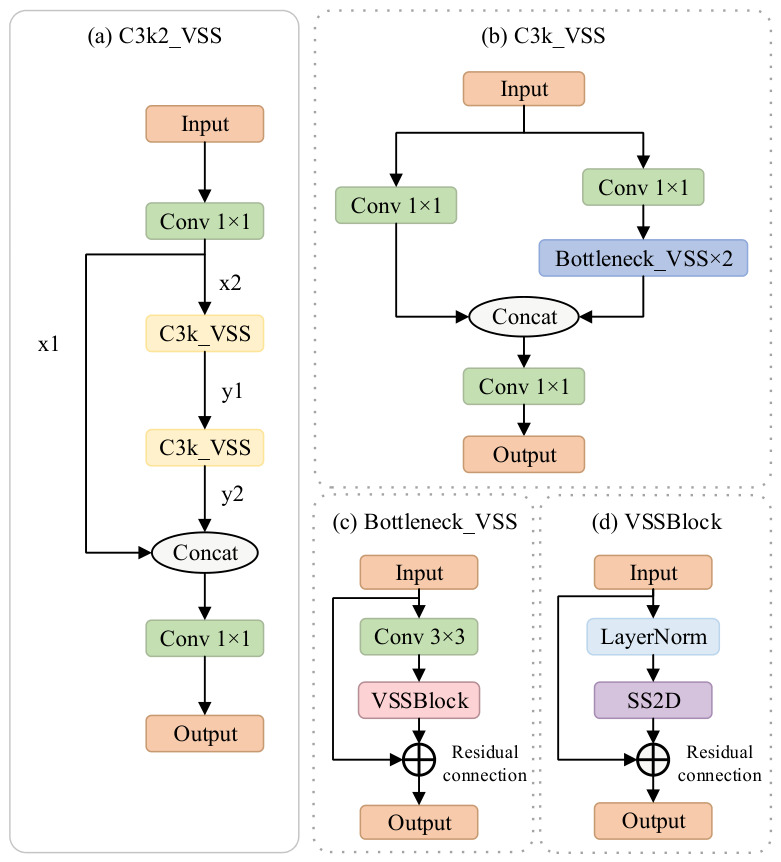
The structures of C3k2_VSS: (**a**) C3k2_VSS module; (**b**) C3k_VSS module; (**c**) Bottleneck_VSS module; (**d**) VSSBlock module.

The C3k2_VSS module retains the cross-stage partial feature reuse strategy (Figure 4a). Input features are first projected through a 1×1 convolution and split into two branches: one preserves shallow local information, while the other is processed by the C3k_VSS module to extract deeper features. The branches are then concatenated along the channel dimension and fused via convolution, enabling the module to capture both local details and global semantic information with controlled parameter size. C3k_VSS employs a dual-branch aggregation structure (Figure 4b), where one branch maintains basic features and the other stacks multiple Bottleneck_VSS modules to enhance feature representation. In Bottleneck_VSS (Figure 4c), an initial convolution extracts local information and adjusts channel dimensions, while VSSBlock models global context, allowing simultaneous learning of local features and long-range dependencies. A residual connection is added when input and output dimensions match to improve gradient propagation and feature reuse. VSSBlock consists of a normalization layer, an SS2D unit (Figure 4d), and a residual connection. SS2D converts two-dimensional (2D) features into sequences along multiple directions, captures global dependencies via state-space recurrent computation, and reconstructs them back into the 2D feature space, thereby enhancing feature representation with low computational overhead.

#### 2.2.2. DySample Upsampling Module

Upsampling quality during multiscale feature fusion directly affects spatial alignment between high- and low-level features and the recovery of object boundaries and local details. Although fixed interpolation is computationally efficient, its sampling positions cannot adapt to image content, often causing blurring around seedling edges and fine details. Therefore, the fixed upsampling operation was replaced with DySample, which improves upsampled feature representation through content-aware dynamic resampling [21].

**Figure 5:**
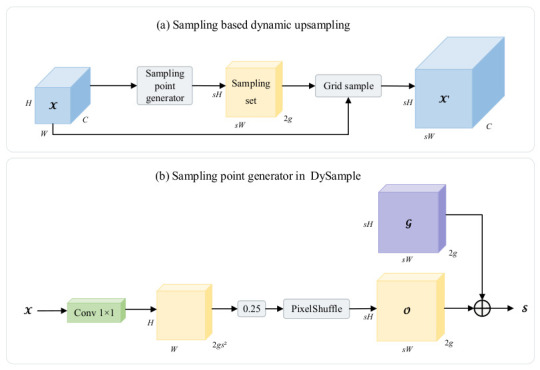
The structure of the DySample module. The input feature, upsampled feature, generated offset, and original grid are denoted by X, $X^{\prime }$, O, and G, respectively: (**a**) The sampling set is generated by the sampling point generator, and the input feature X is re-sampled using the grid_sample function to produce $X^{\prime }$. (**b**) Detailed structure of the sampling point generator under the static scope factor configuration, where the final sampling set is derived from the sum of the generated offset O and the original grid position G.

The DySample module consists of two main components: sampling-point generation and feature resampling (Figure 5a). The module directly learns sampling offsets in the high-resolution spatial domain and uses them to resample low-resolution features. Unlike fixed interpolation, this content-aware resampling strategy avoids the representational limitations of regular sampling positions and better preserves object boundaries and local details.

During sampling-point generation (Figure 5b), the input features are first passed through a 1×1 convolution to obtain offset information. A scaling factor of 0.25 is applied to the intermediate offsets to constrain the displacement range of sampling points and improve training stability. PixelShuffle then redistributes the offsets from the channel dimension to the spatial dimension according to the upsampling factor, enabling low-resolution offset predictions to correspond to high-resolution sampling locations. These locations are combined with a regular sampling grid to form the final sampling set, and the upsampled features are generated through bilinear sampling. This content-adaptive design allows sampling locations to better fit seedling edges, textures, and local structural variations, thereby improving spatial alignment and detail preservation during multiscale feature fusion with limited additional computational cost.

#### 2.2.3. MG-Slide Loss

Visual differences among seedling-stage categories are often subtle, and easy samples may dominate model training. When all samples are assigned equal weights, the model may be biased toward easily classified samples, weakening the learning of boundary and hard samples. The Slide weighting function in YOLO FaceV2 mitigates this bias by assigning piecewise weights according to the relationship between sample quality and a predefined threshold [22]. However, thresholds derived directly from current-batch statistics are susceptible to batchwise fluctuations and may reduce training stability. Inspired by Wise-IoU, which uses historical statistics to stabilize dynamic adjustment [23], MG-Slide Loss was designed to smooth current-batch statistics with historical information while retaining the piecewise weighting strategy of Slide.

For iteration t, the mean quality statistic of positive samples in the current batch is denoted by $Q_{t}$. The momentum coefficient $d_{t}$ and dynamic threshold $\mu_{t}$ are defined as follows:

(1) $$d_{t}=\beta \cdot \left(1-e^{-\frac{t}{\tau }}\right)$$

(2) $$\mu_{t}=d_{t}\cdot \mu_{t-1}+\left(1-d\right)\cdot Q_{t}$$

where $\mu_{t-1}$ denotes the dynamic threshold from the previous iteration, β is the upper bound of the momentum coefficient, and τ is the time-control parameter. To avoid unstable weight assignment caused by an excessively low threshold during early training, a lower-bound constraint is imposed on $\mu_{t}$:

(3) $${\hat{\mu }}_{t}=max\left(\mu_{t},0.2\right)$$

During loss reweighting, a piecewise weighting function is constructed according to the relationship between the sample supervision value y and the dynamic threshold ${\hat{\mu }}_{t}$:

(4) $$f\left(y\right)=\left\{\begin{array}{cc} 1.0\;,\;\; & y\leq {\hat{\mu }}_{t}-0.1\\ e^{1-{\hat{\mu }}_{t}},\;\; & {\hat{\mu }}_{t}-0.1\leq y\leq {\hat{\mu }}_{t}\\ e^{-\left(y-1\right)},\;\; & y\geq {\hat{\mu }}_{t} \end{array}\right.$$

The final classification loss is expressed as:

(5) $$L_{MG-Slide}=f\left(y\right)\cdot L_{BCE}$$

where $L_{BCE}$ denotes the basic binary cross entropy loss. This loss preserves the original weight for low-quality samples, increases the weight of samples near the threshold to emphasize boundary and ambiguous samples, and reduces the weight of high-quality easy samples to suppress redundant gradients. Thus, MG-Slide Loss retains the difficulty-aware weighting mechanism of Slide while reducing batchwise threshold fluctuations, thereby improving model discrimination.

### 2.3. Lightweight VSG-UNet Segmentation Network

**Figure 6:**
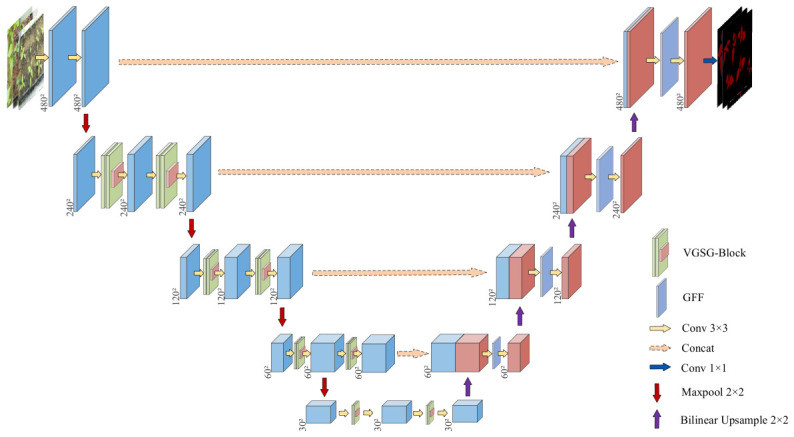
The structures of VSG-UNet.

Accurate leaf segmentation requires reliable leaf-region extraction and boundary recovery under complex background conditions. To balance segmentation accuracy and model efficiency, the original U-Net framework was modified to construct the lightweight VSG-UNet. Figure 6 shows the overall architecture of VSG-UNet. The SG-Block and VGCA modules were introduced into the encoder to enhance texture feature representation, whereas the GFF module was incorporated into the decoder to improve multi-level feature fusion and leaf boundary recovery. The structures and functions of these three modules are described below.

#### 2.3.1. Strip-Ghost Bottleneck

Pepper leaves at the seedling stage are small, and their vein and lamina textures differ along the horizontal and vertical directions. In plug-tray seedling images, substrate particles, tray edges, and wet regions introduce background interference, making small leaf features difficult to distinguish from the surrounding background. Although standard 3×3 convolutions are general-purpose operators, they may aggregate target and background responses simultaneously, leading to redundant feature responses and increased computational cost. Because convolutional feature maps contain redundant information, complementary features can be generated through low-cost operations [24]. Asymmetric convolutions can expand the receptive field along specific directions with limited computational overhead, thereby improving spatial modeling efficiency [25]. Depthwise asymmetric convolutions further combine directional modeling with lightweight feature extraction, making them suitable for efficient feature representation in segmentation networks [26]. Based on this rationale, the Strip-Ghost Bottleneck (SG-Block) module was designed to enhance the representation of seedling leaf contours, vein orientations, and local elongated structures.

**Figure 7:**
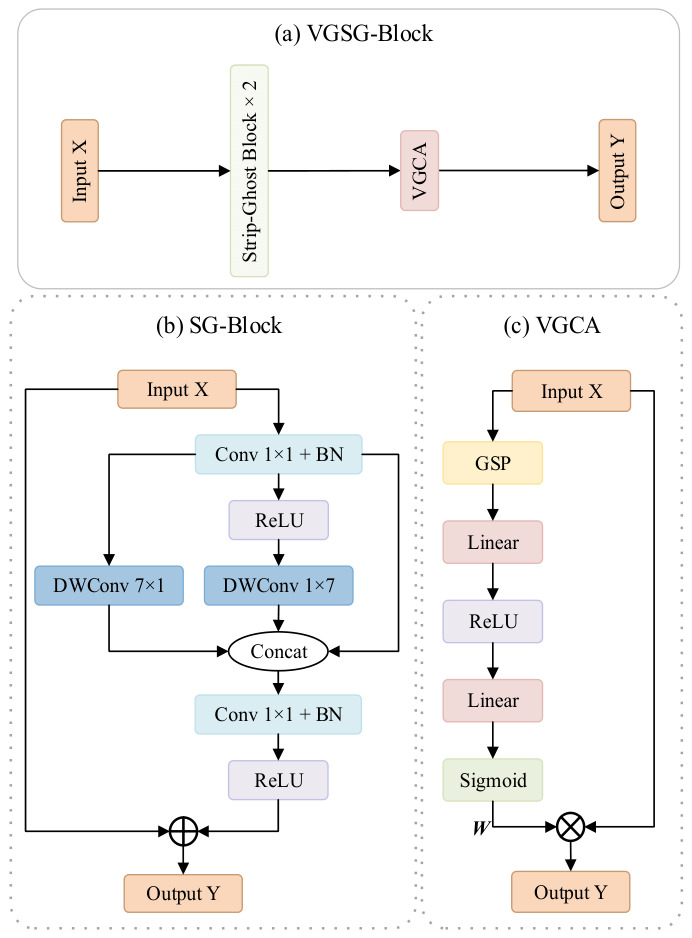
The structures of the VGSG-Block: (**a**) VGSG-Block module; (**b**) SG-Block module; (**c**) VGCA module. The VGCA module is connected after the SG-Block; together, they constitute the VGSG-Block.

The module first applies a 1×1 pointwise convolution to compress the input channels and extract basic features, reducing computational cost while preserving key semantic information (Figure 7b). The compressed features are then fed into three branches: one retains the basic features, whereas the other two use 7×1 and 1×7 depthwise convolutions to extract vertical and horizontal strip features. This design enhances directional structural representation rather than simply replacing standard convolution.

For targets such as pepper leaves at the seedling stage, the vertical branch helps capture longitudinal textures and edge extension information, whereas the horizontal branch helps represent transverse texture variations. Together with the main branch, these two branches form a more comprehensive feature representation. After the three-branch features are concatenated along the channel dimension, a 1×1 pointwise convolution is applied for fusion, producing output features that integrate local texture information with directional structural information. When the input and output have the same number of channels and spatial dimensions, a residual connection is used for element-wise addition to improve feature transmission stability and gradient propagation. While maintaining a lightweight design, this module improves the network’s directional feature extraction capability and provides more effective basic features for subsequent feature fusion and decoding.

#### 2.3.2. Variance-Guided Channel Attention

After SG-Block feature extraction, channel responses to seedling leaves and background regions remain heterogeneous. Channel attention is therefore introduced to recalibrate informative channels by compressing spatial responses into channel descriptors and learning channel-wise importance [27]. However, global average pooling mainly reflects overall response intensity and is less effective in characterizing intra-channel texture variations and local fluctuations. Since richer statistical descriptors can improve channel representation [28], a Variance-Guided Channel Attention (VGCA) module was designed to construct channel descriptors from spatial fluctuation intensity and generate adaptive channel weights (Figure 7c).

For an input feature X, the spatial standard deviation $\sigma_{c}$ corresponding to channel c is computed as follows:

(6) $$\sigma_{c}=\sqrt{\frac{1}{H\times W}\sum_{i=1}^{H}\sum_{j=1}^{W}{\left(X_{c}\left(i,j\right)-\mu_{c}\right)}^{2}+\epsilon }$$

where $X_{c}(i,j)$ represents the feature value at spatial position (i,j) in channel c, and H and W denote the height and width of the feature map, $\mu_{c}$ denotes the spatial mean of channel c, and ϵ is a small constant used to prevent numerical instability. The channel statistical vector is then obtained as $\sigma =\left[\sigma_{1},\sigma_{2},\cdots \sigma_{c}\right]$. Compared with global average pooling, standard deviation captures both response intensity and intra-channel dispersion, making it more sensitive to leaf edges, vein orientations, and target-background differences.

After obtaining σ, VGCA uses two fully connected layers, ReLU activation, and a sigmoid function to generate normalized channel weights:

(7) $$W=Sigmoid\left(W_{2}\cdot \delta \left(W_{1}\cdot \sigma \right)\right)$$

where δ denotes the ReLU activation function, and $W_{1}$ and $W_{2}$ are the parameters of the two fully connected layers. Finally, the generated channel weights W are reshaped into a c×1×1 tensor and multiplied channel-wise with the input feature to obtain the output Y feature:

(8) Y=X⊙W

In this way, VGCA enhances leaf texture and edge information while suppressing interference from smooth background regions and noisy channels. In the network implementation, VGCA is connected after SG-Block (Figure 7a), and together they form the Variance-Guided Strip-Ghost Block (VGSG-Block).

#### 2.3.3. Gated Feature Fusion

During the decoding stage of U-Net, shallow features contain rich edge and texture details, whereas deep features provide stronger semantic information. Although direct concatenation can integrate multilevel information, it is susceptible to semantic discrepancies, resulting in insufficient alignment between fine details and high-level semantics. Conventional skip connections have certain limitations in feature fusion because encoder and decoder features at the same scale are not fully consistent semantically, and direct fusion may not achieve optimal performance [29]. Meanwhile, gating mechanisms can regulate information propagation during fusion and reduce interference from irrelevant information and noise [30]. Based on this rationale, a Gated Feature Fusion (GFF) module was designed to enhance the matching between shallow detail information and deep semantic information in skip connections, thereby improving feature fusion performance.

**Figure 8:**
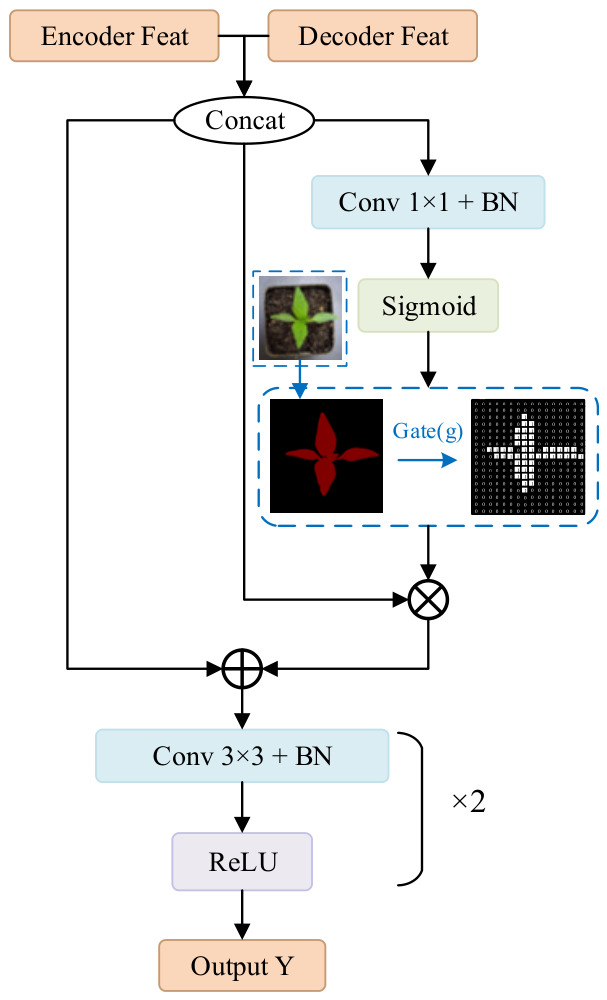
The structures of GFF.

The GFF module first concatenates the upsampled decoder features with the corresponding encoder features, and then generates spatial gating weights through a 1×1 convolution, batch normalization, and a sigmoid function (Figure 8). These weights measure the importance of different spatial locations and adaptively regulate the fused features. Unlike direct concatenation, this strategy enhances responses in target-relevant regions while suppressing background interference. To avoid information loss after gating, a residual fusion strategy combines the weighted features with the original concatenated features, preserving basic information while enhancing salient regions. Finally, two consecutive 3×3 convolutional layers are used to integrate local contextual information and produce a stable output representation. This design improves the correspondence between encoder and decoder features and enhances the representation of leaf edges, small targets, and fine details.

### 2.4. Leaf Area Correction

Leaf inclination was quantified using the angle measured in ImageJ software version 1.8.0 between the main leaf axis, defined as the line from the petiole to the leaf tip, and the horizontal direction. Because ImageJ software reports signed angles relative to the positive *x*-axis within the range of -180° to 180°, the angle was standardized as follows:

(9) $$\theta_{leaf}=min\left(\left|\theta \right|,180{}^{\circ}-\left|\theta \right|\right)$$

where θ denotes the original angle output by ImageJ software, and $\theta_{leaf}$ denotes the standardized leaf inclination angle.

The single-leaf areas measured using ImageJ software were summed for each seedling to obtain the total 2D projected leaf area. Because the measured image area represents the vertical projection of inclined leaves, a cosine-based correction was applied according to the geometric relationship between leaf inclination and projected area [31]:

(10) $$A_{real}=\frac{A_{proj}}{\cos\theta_{leaf}}$$

where $A_{proj}$ denotes the total projected leaf area, and $A_{real}$ denotes the corrected approximate true leaf area. This cosine-based correction provides an approximate compensation for inclination-induced underestimation of projected leaf area and provides adjusted area traits for subsequent phenotypic modeling and leaf dry biomass estimation. However, it assumes that leaves can be simplified as approximately planar surfaces and therefore cannot fully account for leaf curvature, overlap, twisting, self-shading, or non-planar canopy structure.

### 2.5. Environmental Settings

Model training and testing in this study were conducted on a cloud server. The hardware and software configurations are listed in Table 2, and the training parameters are presented in Table 3.

### 2.6. Evaluation Metrics

Evaluation metrics for the VDM-YOLO network include precision, recall, F1-score, and mAP_0.5_. Precision characterizes the reliability of positive predictions and reflects the model’s ability to suppress false detections in complex scenes. Recall measures the completeness of positive sample detection. The F1-score, defined as the harmonic mean of precision and recall, provides a comprehensive assessment of the balance between detection accuracy and completeness. mAP_0.5_ denotes the mean average precision (mAP) at an intersection over union (IoU) threshold of 0.5. These metrics are calculated as follows:

(11) $$P=\frac{TP}{TP+FP}\times 100\%$$

(12) $$R=\frac{TP}{TP+FN}\times 100\%$$

(13) $$F1=\frac{2PR}{P+R}\times 100\%$$

(14) $$AP=\int_{0}^{1}P\left(R\right)dR$$

(15) $${mAP}_{0.5}=\frac{1}{N}\sum_{i=1}^{N}{AP}_{i}\times 100\%\left(IoU\geq 0.5\right)$$

where TP, FP, and FN denote the numbers of true positives, false positives, and false negatives. AP denotes average precision, ${AP}_{i}$ represents the average precision for class i and N denotes the total number of classes.

Evaluation metrics for the VSG-UNet network included pixel accuracy, mean intersection over union (mIoU), and the Dice coefficient. Pixel accuracy was used to characterize the overall pixel-level classification accuracy on the validation set, reflecting the model’s ability to distinguish foreground and background regions. mIoU was used to measure the average segmentation performance across classes and comprehensively quantify the overlap between predicted regions and ground-truth annotations. The Dice coefficient was used to describe the agreement between the predicted foreground region and the ground-truth foreground region, providing a more intuitive evaluation of target-region segmentation performance. These metrics were calculated as follows:

(16) $$PA=\frac{N_{correct}}{N_{total}}$$

(17) $$IoU=\frac{\left|X\cap Y\right|}{\left|X\cup Y\right|}$$

(18) $$mIoU=\frac{{IoU}_{leaf}+{IoU}_{background}}{2}$$

(19) $$Dice=\frac{2\left|X\cap Y\right|}{\left|X\right|+\left|Y\right|}$$

where $N_{correct}$ denotes the number of correctly classified pixels, and $N_{total}$ denotes the total number of pixels. X represents the predicted foreground region, whereas Y represents the ground truth foreground region. ${IoU}_{leaf}$ and ${IoU}_{background}$ denote the intersection over union values for the foreground and background classes.

For the independent leaf dry weight validation set, uncertainty in *R*^2^, RMSE, and mean absolute error (MAE) was estimated using paired nonparametric bootstrap resampling. Specifically, the measured and predicted leaf dry weight values were resampled together 10,000 times with replacement, and the 2.5th and 97.5th percentiles of the resulting metric distributions were used as the 95% confidence intervals (CIs). Because the validation set was limited in size, these CIs were used to describe the uncertainty of the preliminary validation metrics rather than to imply broad external generalizability.

## 3. Results

### 3.1. Comparison of Different Detection Networks

To evaluate the applicability of different object detection networks for identifying the stage of growth and localizing pepper seedlings, several mainstream networks based on YOLO were compared. All networks were trained and tested on the same dataset under identical experimental conditions. The results are presented in Table 4.

YOLOv5, YOLOv8, YOLOv9, YOLOv10, and YOLOv11 all yielded lower precision, F1-score, and mAP_0.5_ than YOLOv12. Mamba-YOLO improved precision and F1-score slightly, but provided only a limited gain in mAP_0.5_, while substantially increasing parameters and computational cost. In contrast, VDM-YOLO achieved a precision of 86.59%, an F1-score of 84.09%, and an mAP_0.5_ of 89.27%, exceeding YOLOv12 by 3.83, 2.11, and 1.82 percentage points, respectively. Although the mAP_0.5_ gain was modest, the concurrent improvements in precision and F1-score suggest that the proposed model improved the reliability of seedling localization and growth-stage recognition under complex greenhouse backgrounds. However, because statistical significance testing was not performed for the differences among detection models, these performance improvements should be interpreted as descriptive comparisons under the current experimental conditions rather than as statistically confirmed differences. These results indicate that VDM-YOLO tended to improve prediction reliability by reducing false detections, but this was accompanied by a slight reduction in recall compared with some YOLO variants. In the proposed cascaded framework, higher precision helps avoid introducing background or non-seedling regions into ROI cropping, which is beneficial for subsequent leaf segmentation and leaf dry biomass estimation. However, for applications requiring complete seedling coverage, such as large-scale nursery monitoring, the detection configuration should be further optimized to balance false detections and missed seedlings according to the target management scenario.

### 3.2. Ablation Experiment Results of VDM-YOLO

To assess the contribution of each improved module to identifying the stage of growth and localizing pepper seedlings, ablation experiments were performed on the Detection dataset using YOLOv12 as the baseline network. The corresponding experimental results are summarized in Table 5.

Adding C3k2_VSS alone improved precision to 83.73%, F1-score to 83.13%, and mAP_0.5_ to 88.44%, indicating that visual state space modeling enhanced global semantic representation and improved seedling discrimination in complex backgrounds. Adding DySample alone increased precision to 84.88% and reduced the parameters to 2.37 M and computational cost to 5.6 G FLOPs, suggesting that content-aware dynamic resampling improved spatial alignment during multiscale feature fusion while maintaining model efficiency. Adding MG-Slide Loss alone improved precision to 83.90%, F1-score to 82.92%, and mAP_0.5_ to 88.99%, reflecting its ability to emphasize easily confused and hard samples through dynamic loss reweighting. However, the ablation results also reveal trade-offs among different module combinations. When C3k2_VSS and MG-Slide Loss were combined, precision increased to 84.96%, but recall decreased to 82.90% and F1-score was 82.31%, suggesting that stronger contextual representation and hard sample reweighting made the model more selective for ambiguous targets. Similarly, the combination of C3k2_VSS and DySample achieved high precision of 86.04% and mAP_0.5_ of 89.01%, but recall decreased to 82.96%, indicating that improved localization reliability was accompanied by a higher risk of missed detections. In contrast, combining DySample and MG-Slide Loss increased recall to 85.56% and mAP_0.5_ to 88.79%, but precision was lower than that of the final model, suggesting that this combination retained more target seedlings but was less effective in suppressing false positives. With all three modules incorporated, the model achieved precision of 86.59%, recall of 84.76%, F1-score of 84.09%, and mAP_0.5_ of 89.27%. Although the recall was not the highest among all ablation settings, the final configuration provided the best F1-score and mAP_0.5_, indicating a more favorable balance between false positive suppression and missed detection reduction. Although parameters and FLOPs increased slightly compared with the baseline, the increase remained limited, indicating that the improved model achieved a favorable balance between detection performance and computational cost.

**Figure 9:**
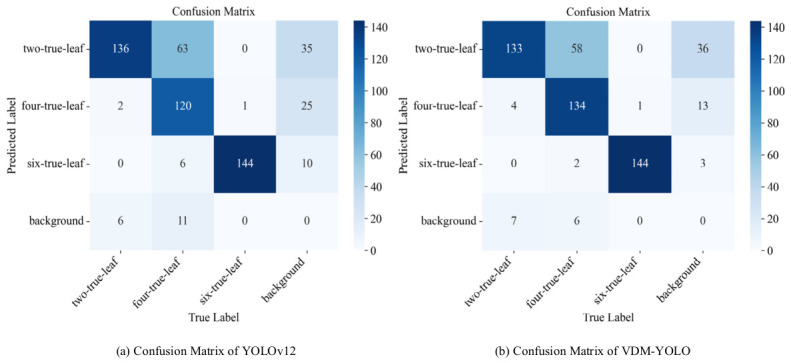
Confusion matrices of YOLOv12 and VDM-YOLO: (**a**) confusion matrix of YOLOv12; (**b**) confusion matrix of the proposed VDM-YOLO.

Compared with the YOLOv12 (Figure 9a), the proposed VDM-YOLO (Figure 9b) produced more concentrated recognition results across most categories, effectively reducing background false detections and inter-stage misclassifications. For the four-true-leaf stage, correctly identified samples increased markedly from 120 to 134, while misclassifications as background decreased from 11 to 6, indicating substantially improved discrimination of seedlings at the intermediate growth-stage. Although correctly classified two-true-leaf samples slightly decreased from 136 to 133, the six-true-leaf stage remained stable in performance, with fewer misclassifications as earlier stages or background, reflecting stronger separation of later-stage seedlings from complex backgrounds.

### 3.3. Comparison of Different Segmentation Networks

To evaluate the applicability of different segmentation networks for leaf segmentation of pepper seedlings, several commonly used semantic segmentation networks were compared. All networks were evaluated on the same dataset under identical training conditions. The results are presented in Table 6.

Among the comparison networks, U-Net outperformed DeepLabV3, FCN, and U^2^-Net, achieving an mIoU of 82.40%, a Dice coefficient of 76.60%, and a PA of 94.60%. VSG-UNet achieved the best performance, with an mIoU of 83.90%, a Dice of 81.80%, and a PA of 95.00%, exceeding U-Net by 1.50%, 5.20%, and 0.40%. In terms of model complexity, VSG-UNet required 78.81 G FLOPs, lower than DeepLabV3, FCN, U^2^-Net, and U-Net, and contained only 6.70 M parameters, substantially fewer than the other networks. These results indicate that VSG-UNet achieved a modest improvement in mIoU compared with U-Net, while showing clearer advantages in Dice coefficient and model efficiency. Therefore, the main benefit of VSG-UNet lies in improving foreground overlap and leaf-region integrity while substantially reducing computational complexity, rather than producing a large increase in overall segmentation accuracy.

**Figure 10:**
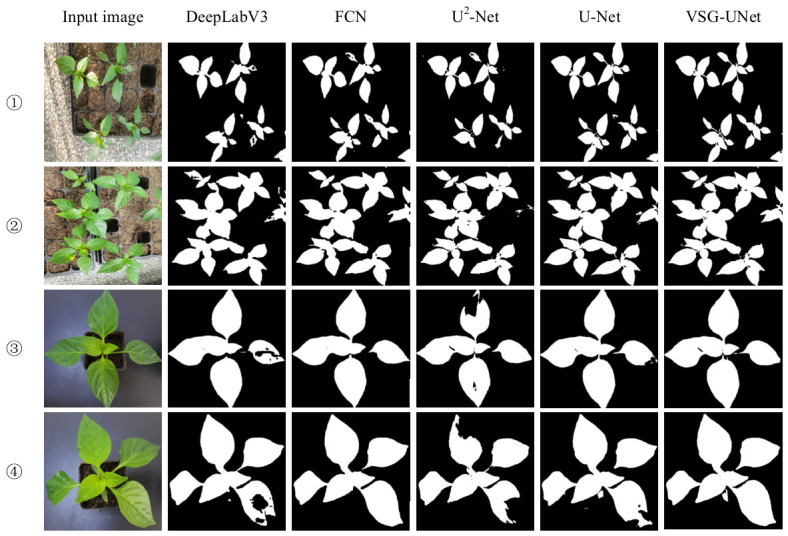
Predicted masks generated by different segmentation models. The labels ➀–➃ indicate different representative samples.

All networks extracted the main seedling leaf regions, but clear differences were observed in small-leaf recognition, boundary preservation, and background suppression (Figure 10). DeepLabV3 and FCN were more prone to missing small leaves, leaf tips, and leaf junctions, with relatively coarse boundaries. U^2^-Net and U-Net improved leaf integrity, but detail recovery remained limited. Visual comparison of the predicted masks further showed that VSG-UNet preserved small leaves and overlapping regions more completely, produced clearer leaf boundaries, and reduced background interference more effectively than the comparison models.

### 3.4. Ablation Experiment Results of VSG-UNet

To evaluate the contribution of each improved module to leaf segmentation of pepper seedlings, ablation experiments were performed on the Segmentation dataset, with U-Net adopted as the baseline model. The corresponding results are presented in Table 7.

Without the proposed modules, the U-Net baseline achieved an mIoU of 82.4%, a Dice coefficient of 76.6%, and a PA of 94.6%, requiring 141.29 G FLOPs and 17.26 M parameters. Adding SG-Block alone slightly improved mIoU to 82.7% and PA to 94.7%, while reducing FLOPs to 78.75 G and parameters to 6.39 M, indicating its effectiveness in lightweight feature extraction. Adding GFF alone improved mIoU to 83.1% and Dice to 78.7%, but retained relatively high computational complexity, with 109.47 G FLOPs and 13.39 M parameters, suggesting that GFF primarily enhances feature fusion rather than model compression. Combining SG-Block and GFF further improved mIoU to 83.7% and Dice to 81.2%, while maintaining 78.81 G FLOPs and 6.39 M parameters. With all three modules incorporated, the model achieved the best performance, with an mIoU of 83.9%, a Dice coefficient of 81.8%, and a PA of 95.0%. Compared with the baseline, FLOPs and parameters were reduced by approximately 44.2% and 61.2%, respectively, demonstrating that the proposed method improves segmentation performance while substantially reducing model complexity.

### 3.5. Analysis of Segmentation Based on Cropped Localization

To further verify the improvement in seedling leaf segmentation achieved by cropping based on localization, one representative seedling was selected from each of the three growth stages, and the segmentation results with and without this cropping step were visually compared.

**Figure 11:**
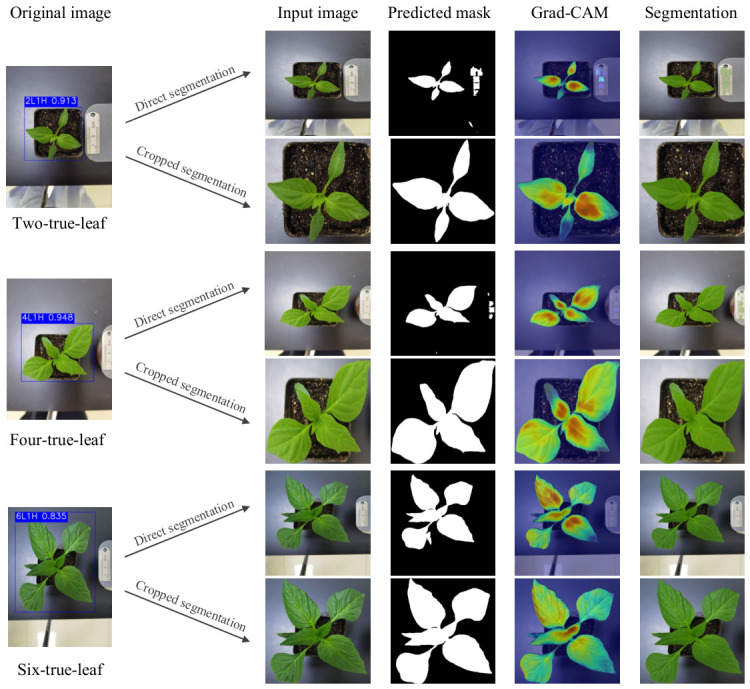
Comparison of segmentation results before and after localization cropping. The annotations “2L1H”, “4L1H”, and “6L1H” in the detection boxes correspond to the two-, four-, and six-true-leaf growth stages, respectively.

Direct segmentation captured the main leaf regions but was affected by large background areas, low target proportion, and visual interference, resulting in incomplete segmentation of small leaves, leaf margins, and junctions (Figure 11). Following the use of localization for cropping, the seedling regions became more prominent and background redundancy was reduced, producing more complete masks, clearer boundaries, and better agreement with true leaf contours. This improvement was most evident at the two- and four-true-leaf stages, where cropping improved the preservation of small leaves and local boundaries. At the six-true-leaf stage, it also produced more focused responses and smoother boundary recovery. Overall, cropping based on localization improved segmentation completeness and stability by reducing irrelevant background information and increasing the relative proportion of target regions.

### 3.6. Phenotypic Segmentation Correction

**Figure 12:**
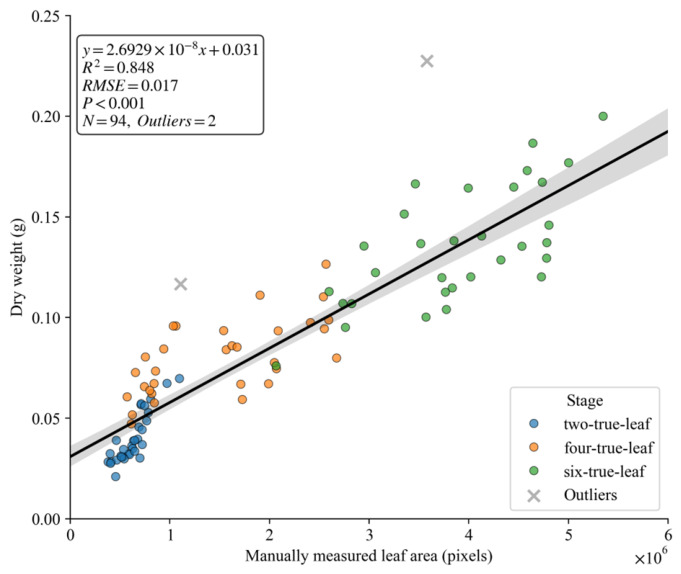
Linear regression between leaf area and leaf dry weight. The shaded area represents the 95% confidence band of the fitted regression line.

**Figure 13:**
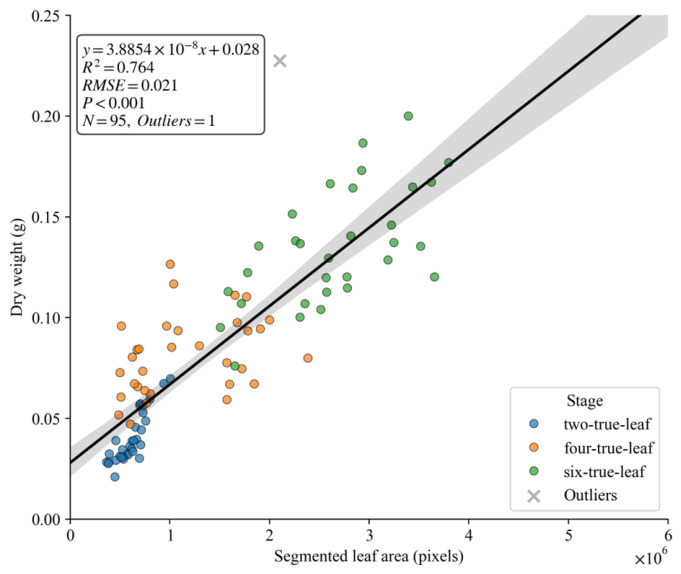
Linear regression between segmented leaf area and leaf dry weight. The shaded area represents the 95% confidence band of the fitted regression line.

The data used for phenotypic segmentation correction were obtained from 96 individual seedlings, with 32 seedlings selected from each of the three seedling stages. Because biological measurements are prone to variability, outliers were screened using a robust modified Z-score method based on the median and median absolute deviation (MAD) [32]. For a given data point $x_{i}$, the modified Z-score is defined as follows:

(20) $$M_{i}=\frac{0.6745\left(x_{i}-\bar{x}\right)}{MAD}$$

where $\bar{x}$ denotes the median of the sample data, and MAD denotes the median absolute deviation. Samples with $\left|M_{i}\right|>3.5$ were identified as outliers and removed [33]. After outlier removal, the manually measured true leaf area showed strong agreement with leaf dry weight, with an *R*^2^ of 0.848 and a RMSE of 0.017 g (Figure 12). In contrast, the VSG-UNet segmented area showed weaker agreement with leaf dry weight, with an *R*^2^ of 0.764 and an RMSE of 0.0210 g (Figure 13), indicating a systematic bias that required further correction.

**Figure 14:**
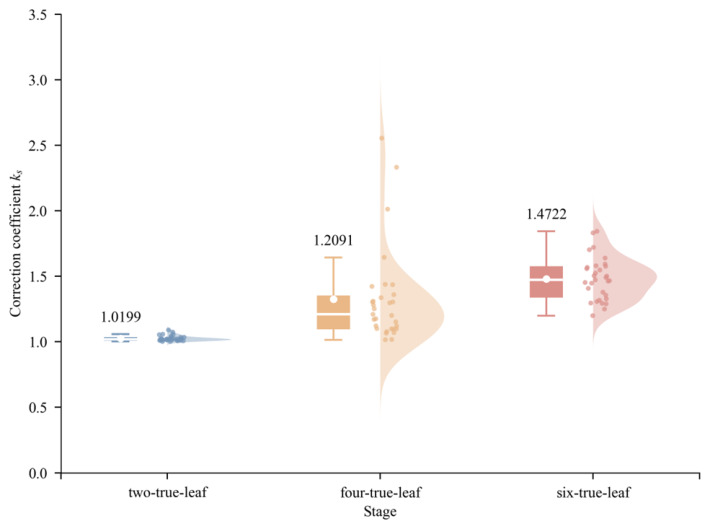
Distribution of the proportional correction coefficient $k_{s}$ across growth stages.

**Figure 15:**
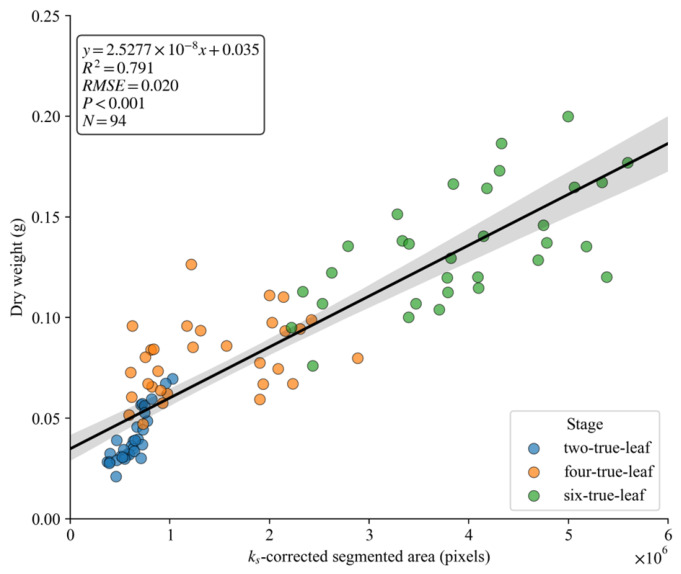
Linear regression between leaf area and leaf dry weight after $k_{s}$ correction. The shaded area represents the 95% confidence band of the fitted regression line.

To reduce this bias, a stage-aware proportional correction coefficient $k_{s}$ was calculated using the median ratio of manually measured leaf area to segmented area within each seedling stage:

(21) $$A_{k}^{s}=k_{s}\cdot A_{mod}^{s}$$

where s denotes the seedling stage. The $k_{s}$ values for the two-, four-, and six-true-leaf stages were 1.0199, 1.2091, and 1.4722, respectively (Figure 14). After proportional correction, the fitting performance improved moderately, with *R*^2^ increasing to 0.791 and RMSE decreasing to 0.0200 g (Figure 15). However, the limited improvement suggested that proportional correction reduced scale bias but could not fully eliminate fixed bias across growth stages. Therefore, a global scaling coefficient a and a stage-aware intercept $c_{s}$ were further introduced to construct the final area correction model:

(22) $$A_{corr}^{s}=a\times \left(k_{s}\cdot A_{mod}^{s}\right)+c_{s}$$

**Figure 16:**
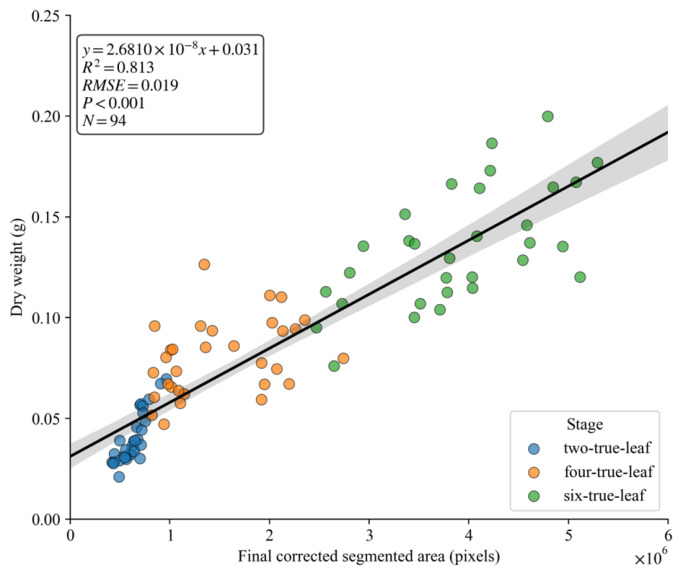
Linear regression between leaf area and leaf dry weight after final correction. The shaded area represents the 95% confidence band of the fitted regression line.

The global scaling coefficient a was 0.836786, and the stage-aware intercepts $c_{s}$ for the two-, four-, and six-true-leaf stages were 106,063.08, 328,527.71, and 610,572.34, respectively. After final correction, the agreement between segmented area and leaf dry weight further improved, with *R*^2^ increasing to 0.813 and RMSE decreasing to 0.0190 g (Figure 16). Compared with the uncorrected results, *R*^2^ increased by 0.049 and RMSE decreased by 0.0020 g. These results indicate that the stage-aware phenotypic segmentation correction produced a moderate and potentially useful improvement. The main value of this correction is not only the numerical increase in *R*^2^, but also the reduction in growth-stage-related systematic bias in the relationship between segmented projected area and leaf dry biomass. This is important for seedling phenotyping because seedlings at different true leaf stages differ in leaf inclination, overlap, canopy compactness, and projected area to biomass relationships. Therefore, incorporating growth-stage information makes the image-derived area trait more comparable across developmental stages and helps limit error propagation from leaf segmentation to leaf dry biomass estimation.

### 3.7. Estimation of Leaf Dry Weight from Corrected Leaf Area

To provide an independent preliminary evaluation across growth stages, 15 seedlings not used for correction model construction were selected as an independent validation set, including five validation plants from each growth stage, namely the two-, four-, and six-true-leaf stages. The model segmented area was corrected using the stage-aware area correction formula and then used as the predictor variable in the established fitting equation for leaf dry biomass estimation. The estimated leaf dry weight was compared with the measured leaf dry weight to evaluate the feasibility of nondestructive estimation across the three true leaf stages.

**Figure 17:**
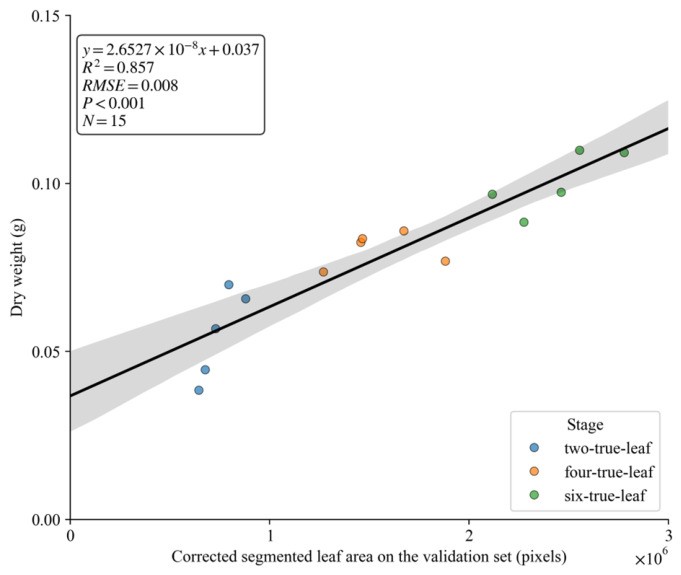
Linear regression between corrected area and measured leaf dry weight based on the validation set. The shaded area represents the 95% confidence band of the fitted regression line.

**Figure 18:**
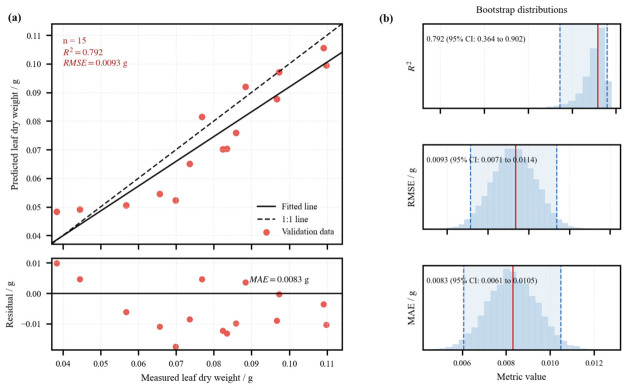
Comparison between measured and predicted leaf dry weight based on the independent validation set. (**a**) Relationship between measured and predicted leaf dry weight and residual distribution. The dashed line indicates the 1:1 reference line. (**b**) Bootstrap distributions and 95% confidence intervals for *R*^2^, RMSE, and MAE based on 10,000 paired resamples. The red vertical lines indicate the point estimates, the blue dashed vertical lines indicate the 95% confidence interval limits, and the light blue shaded areas indicate the corresponding 95% confidence intervals.

The corrected area showed an apparent linear relationship with measured leaf dry weight in the independent validation set, with an *R*^2^ of 0.857 and an RMSE of 0.0080 g (Figure 17). Samples from the three growth stages followed a similar regression trend, suggesting preliminary cross-stage applicability of the correction model under the tested conditions. After the corrected area was used for leaf dry weight prediction, the predicted and measured values in the independent validation set of 15 seedlings showed reasonable agreement, with an *R*^2^ of 0.792 (95% CI: 0.364 to 0.902), an RMSE of 0.0093 g (95% CI: 0.0071 to 0.0114 g), and an MAE of 0.0083 g (95% CI: 0.0061 to 0.0105 g) (Figure 18). The relatively wide confidence interval for *R*^2^ reflects the limited size of the independent validation set and indicates that the biomass prediction performance should be interpreted cautiously. Most samples were distributed near the 1:1 reference line, although residual deviations remained, likely due to individual morphological differences and within-stage growth variability. Overall, the proposed area correction and leaf dry weight prediction method provided preliminary evidence, with quantified uncertainty, that corrected image-derived leaf area may be useful for nondestructive leaf dry weight prediction across the three true-leaf stages under the tested greenhouse conditions.

## 4. Discussion

The proposed framework addresses pepper seedling phenotyping by dividing the task into three connected but functionally distinct steps. First, seedling localization reduces redundant background information from plug trays, substrate, and neighboring plants before leaf segmentation. Second, ROI-based segmentation allows VSG-UNet to focus on leaf regions at a finer spatial scale, which improves boundary preservation and reduces background interference. Third, the incorporation of growth-stage information helps correct the developmental bias between two-dimensional projected leaf area and leaf dry biomass, because leaf inclination, overlap, canopy compactness, and dry matter accumulation change during seedling development.

In the detection module, VDM-YOLO provided seedling localization and growth-stage information for the downstream segmentation and leaf dry biomass estimation steps. Compared with previous YOLO-based pepper detection and growth-stage recognition studies that mainly focused on larger plants or more visually distinct developmental stages, the present study addressed smaller plug tray seedlings with subtler interstage differences and stronger background interference. The necessity of the three VDM-YOLO components can therefore be understood from these phenotypic challenges rather than from numerical ablation gains alone. The VSS component improves global contextual representation, helping distinguish small seedlings from tray cells, substrate regions, shadows, and neighboring plants. DySample improves spatial alignment during multiscale feature fusion, which helps preserve seedling boundaries when leaves are small, inclined, or partially overlapping. MG-Slide emphasizes ambiguous and hard samples during training, which is relevant because the visual differences among the two-, four-, and six-true-leaf stages are subtle. Therefore, although some module combinations produced limited or non-monotonic improvements, the three components address complementary challenges related to background interference, boundary localization, and subtle growth-stage discrimination. In this context, reliable localization is not only an object detection task but also a preprocessing step that determines the quality of subsequent ROI cropping and leaf segmentation.

For the segmentation component, VSG-UNet achieved a favorable balance between segmentation performance and lightweight architecture. Rather than relying mainly on increased model size, the network was designed to maintain stable leaf segmentation performance while reducing model size and computational cost. This is important for plug tray seedling phenotyping, where large background areas, small leaves, and overlapping leaf regions can reduce the efficiency and stability of direct full-image segmentation. Because the segmentation model is applied after ROI cropping and may need to process many individual seedlings in batch nursery phenotyping, a compact model with comparable or slightly improved segmentation accuracy can be more suitable for practical workflows than a larger model with only marginally higher accuracy. Therefore, the limited increases in mIoU and pixel accuracy indicate that VSG-UNet provides incremental segmentation improvements while improving model compactness and computational efficiency within the proposed phenotyping pipeline.

**Figure 19:**
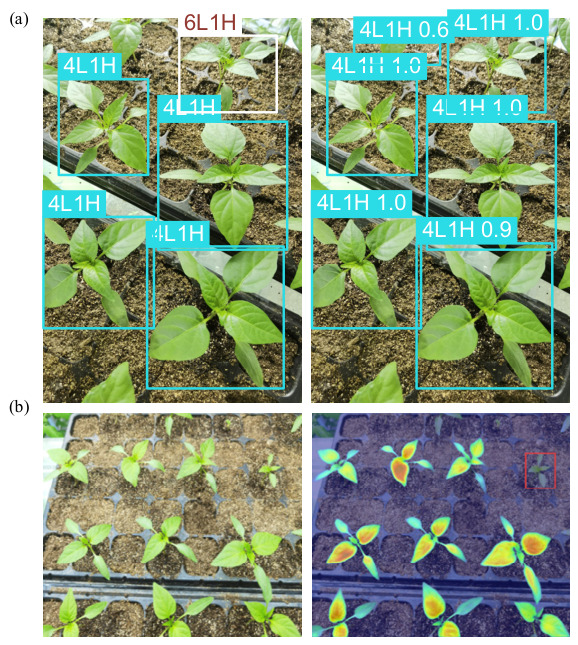
Representative failure cases of the proposed cascaded framework. (**a**) Detection results for a multi-seedling plug tray image. The left image shows the ground-truth labels, and the right image shows the prediction results. (**b**) Missed segmentation case for a small seedling in a plug tray image. The left image shows the original RGB image, and the right image shows the segmentation result, where the red box indicates the missed small seedling.

The detection and segmentation examples in Figure 19 further illustrate the conditions under which the cascaded framework may become less stable. In the detection stage, the comparison between ground-truth labels and prediction results shows that the six-true-leaf stage can be misclassified as the four-true-leaf stage in multi-seedling plug tray images (Figure 19a). This result is consistent with the confusion matrix, in which the four- and six-true-leaf stages showed relatively higher confusion. The misclassification may be related to the subtle morphological differences between adjacent developmental stages, especially when newly emerged leaves are small, partially occluded, or visually similar to unfolded leaves from earlier stages. Background clutter from substrate particles and tray-cell boundaries, neighboring seedlings, partial leaf overlap, and variation in seedling size may further increase the difficulty of seedling localization and developmental-stage classification. In the segmentation stage, missed segmentation occurred for a small seedling in the plug tray image (Figure 19b), suggesting that small leaves, weak leaf-background contrast, background clutter, and uneven illumination can reduce segmentation stability and increase the risk of incomplete masks. These examples indicate that additional samples representing difficult cases and stratified error analysis across growth stages, leaf size classes, overlap levels, background complexity, and illumination conditions may further improve the robustness of detection, developmental-stage classification, and leaf segmentation.

The stage-aware correction results further indicate that projected leaf area is not a constant proxy for leaf dry biomass during seedling development. Changes in leaf inclination, overlap, canopy compactness, and leaf expansion alter the relationship between segmented area and dry matter accumulation, leading to stage-dependent bias. Therefore, incorporating growth-stage information is necessary for reducing this bias and improving image-based leaf dry biomass estimation.

The moderate improvement after correction also reflects the fact that leaf dry biomass estimation is determined by multiple morphological and physiological factors, whereas the present correction model was based mainly on two-dimensional projected area, leaf inclination, and growth stage. Even under fixed imaging conditions, projected leaf area can be affected by leaf overlap, mutual occlusion, leaf curvature, self-shading, canopy height differences, and non-planar leaf surfaces. In addition, traits such as leaf thickness, dry matter concentration, greenness or chlorophyll related status, and individual growth variation may also influence leaf dry weight estimation. Therefore, the stage-aware correction should be interpreted as a practical bias reduction strategy within an RGB image-based phenotyping framework, rather than as a complete leaf dry weight estimation model incorporating all morphological and physiological traits.

Beyond the biological limitations of the correction model, the validation conditions also constrain the practical generalizability of the framework. The current validation covers controlled greenhouse images with realistic background interference, including plug tray edges, substrate regions, shadows, and neighboring seedlings, but it does not fully represent the variability of commercial nursery production. Under true commercial tray density, stronger leaf overlap and canopy contact may reduce seedling separability and make ROI cropping less reliable. Non-uniform seedlings, mixed cultivar production, disease symptoms, water stress, and nutrient stress may further change leaf size, shape, color, posture, and growth-stage appearance, thereby affecting both detection and segmentation. These conditions may also alter the relationship between projected leaf area and leaf dry biomass because stress and cultivar differences can change leaf thickness, dry matter concentration, and canopy architecture.

This limited scope is also evident when the present framework is compared with recent seedling quality studies. Ha et al. [17] showed that projected canopy area and vegetation indices provide complementary morphological and physiological information for basil seedling evaluation. Tsaniklidis et al. [18] further showed in cucumber seedlings that area estimation at the leaf and whole seedling scales can be combined with greenness or SPAD-related indicators associated with chlorophyll status, vigor, and marketable quality. In contrast, the present work focused on individual seedling localization, ROI-based leaf segmentation, growth-stage recognition, and stage-aware area correction under complex pepper plug tray backgrounds. Thus, the proposed framework provides a basis for morphology and biomass-related phenotyping, while physiological and quality-related traits remain necessary for comprehensive commercial seedling assessment.

Compared with recent three-dimensional phenotyping approaches, such as point cloud reconstruction [34,35], RGB-D imaging [36,37], multi-view reconstruction [38,39], and 3D Gaussian based methods [40], the proposed RGB-based framework is more accessible and less costly, but it cannot directly capture canopy depth, leaf curvature, or complex three-dimensional architecture. Therefore, this method may be suitable for accessible RGB-image-based nursery monitoring under relatively controlled greenhouse imaging conditions, whereas more structurally complex scenes may require additional multi-view or depth information.

For practical nursery deployment, the proposed framework would need to be adapted to the physical structure and management workflow of each greenhouse. The position, number, and viewing angle of RGB cameras should be determined according to seedling bench layout, plug tray size, aisle width, greenhouse span, available mounting points, and whether images are collected by fixed overhead cameras, a moving gantry, a mobile cart, or a conveyor- or bench-mounted imaging station. In a potential workflow, tray-level images could be acquired during routine seedling inspection and then processed in batches to localize individual seedlings, segment leaf regions, identify growth stages, and estimate leaf dry biomass. Commercial application would also require standardized camera height and scale calibration, stable illumination or illumination correction, and automated batch processing. In addition, image acquisition speed, labor requirements for image collection and system maintenance, hardware and installation cost, and robustness under changing greenhouse light conditions should be further evaluated. However, because the present validation was conducted using a single cultivar, greenhouse environment, smartphone imaging device, and experimental season, the current results should be interpreted as proof-of-concept evidence rather than as direct evidence of readiness for operational deployment.

The methodology presented in this study still has certain limitations. First, the cascaded structure may propagate errors from detection to downstream segmentation and leaf dry biomass estimation, which may reduce the overall accuracy of the phenotyping pipeline. Second, the present model was developed and validated using one pepper cultivar, three true-leaf stages, one greenhouse environment, one smartphone imaging device, one experimental season, and a limited independent validation set. Differences in cultivar morphology, greenhouse structure, tray background, illumination regime, seasonal growth conditions, camera optics, image resolution, and imaging distance may affect seedling localization, leaf segmentation, growth-stage recognition, and the relationship between projected leaf area and leaf dry biomass. Moreover, the independent biomass validation set contained only 15 seedlings, and although bootstrap confidence intervals were reported to quantify the uncertainty of *R*^2^, RMSE, and MAE, these estimates cannot replace validation using larger independent datasets. Therefore, the reported biomass prediction performance and overall framework applicability should be regarded as preliminary. Broader external validation using larger and more diverse datasets across cultivars, production seasons, greenhouse systems, management conditions, stress conditions, and imaging devices is necessary before the framework can be recommended for operational deployment in commercial nursery production. Third, although the proposed framework showed reduced or moderate model complexity according to FLOPs and parameter counts, the present study evaluated computational efficiency mainly based on these indirect indicators rather than standardized deployment benchmarks. In addition, the performance differences among detection and segmentation models were reported as descriptive comparisons, and formal statistical significance testing of metrics such as mAP_0.5_, F1-score, mIoU, Dice coefficient, and PA was not conducted.

Future work should incorporate multi-view or 3D imaging, as well as color or greenness-related indicators, to improve the morphological and physiological characterization of pepper seedlings. Further studies should also expand validation across cultivars and environments; use repeated training runs, cross-validation, bootstrap-based comparisons, or permutation-based comparisons to quantify the statistical reliability of observed model-performance differences; reduce dependence on pixel-level annotation; explore more integrated end-to-end optimization between detection, segmentation, and leaf dry biomass estimation modules; incorporate uncertainty estimation to identify unreliable intermediate outputs; and further assess inference latency, peak memory consumption, and hardware compatibility on nursery-compatible platforms, such as embedded GPUs, industrial computers, or compact vision systems, to verify the practical deployment potential of the framework in commercial seedling production. Quantitative error analysis stratified by growth stage, overlap level, and background complexity was not fully performed in the present study because overlap severity and background complexity were not assigned quantitative annotation categories during dataset construction. Therefore, stratified detection and segmentation error analysis across growth stages, leaf size classes, overlap levels, and background complexity levels should be conducted, and additional commercial seedling quality traits such as compactness, uniformity, leaf color, leaf damage, and physiological status should be incorporated to more comprehensively evaluate model robustness and seedling quality in heterogeneous nursery scenes.

## 5. Conclusions

This paper presents a proof-of-concept framework for nondestructive phenotyping of pepper seedlings under restricted greenhouse experimental conditions. By integrating VDM-YOLO, VSG-UNet, and a stage-aware area correction model, the proposed cascaded framework enabled seedling localization, growth-stage classification, leaf segmentation, area correction, and preliminary leaf dry biomass estimation from RGB images. VDM-YOLO achieved a precision of 86.59%, an F1 score of 84.09%, and an mAP_0.5_ of 89.27%, suggesting improved detection performance under the tested conditions and providing regions of interest for subsequent segmentation. VSG-UNet achieved an mIoU of 83.9%, a Dice coefficient of 81.8%, and a PA of 95.0%, while reducing floating point operations and parameters by 44.2% and 61.2%, respectively, indicating a favorable balance between segmentation performance and model complexity. The stage-aware correction method reduced the growth-stage-dependent bias between two-dimensional projected leaf area and leaf dry biomass; after correction, the *R*^2^ between model-segmented area and leaf dry weight increased to 0.813, while RMSE decreased to 0.0190 g. In the independent validation set, the corrected area achieved an *R*^2^ of 0.857, and the fit between measured and predicted leaf dry weight reached an *R*^2^ of 0.792, providing initial evidence for the feasibility of nondestructive leaf dry weight prediction in pepper seedlings. Overall, under the tested greenhouse conditions, this study provides a proof-of-concept quantitative phenotyping framework that links RGB image-based detection, leaf segmentation, stage-aware area correction, and leaf dry biomass estimation. The framework should therefore be regarded as a proof-of-concept rather than a deployment-ready system, and broader external validation together with deployment-oriented testing is still required before operational use in commercial nursery production.

## Figures and Tables

**Table 1:** Dataset composition and sample size.

Dataset	Train	Test	Validation	Training Data Augmentation	Total After Augmentation
Detection	597	172	82	3582	3836
Segmentation	469	0	117	1876	1993

**Table 2:** Hardware configuration and software environment.

Item	Configuration
GPU	NVIDIA GeForce RTX 4090 × 1
GPU Memory	24 GB
CPU	AMD EPYC 7J13 64-Core Processor
System Memory	20 GB
Data Disk	50 GB NVMe
Operating System	Linux
Python Version	Python 3.8
Deep Learning Framework	PyTorch 2.9.1
CUDA Version	CUDA 11.8.0

**Table 3:** Training parameters.

Parameters	VDM-YOLO	VSG-UNet
epochs	200	150
image size	640	480
batch size	32	32
optimizer	SGD	SGD
learning rate	0.01	0.01
momentum	0.937	0.9
weight decay	0.0005	0.0001

**Table 4:** Comparative experimental results of different detection networks.

Networks	P/%	R/%	F1/%	mAP_0.5_/%	FLOPs/G	Params/M
YOLOv5	79.31	85.93	81.07	86.46	7.10	2.50
YOLOv8	77.51	86.49	80.38	87.28	8.10	3.01
YOLOv9	80.71	85.57	80.85	87.60	7.60	1.97
YOLOv10	81.29	83.95	81.57	87.27	6.50	2.27
YOLOv11	81.73	85.21	81.97	86.63	6.30	2.58
Mamba YOLO	83.73	85.00	82.48	86.65	13.60	5.98
YOLOv12	82.76	85.41	81.98	87.45	6.30	2.56
VDM-YOLO	86.59	84.76	84.09	89.27	6.60	3.77

**Table 5:** Ablation experimental results of VDM-YOLO.

Module			P	R	F1	mAP_0.5_	FLOPs	Params
VSS	DySample	MG-Slide						
-	-	-	82.76	85.41	81.98	87.45	6.30	2.56
√	-	-	83.73	85.31	83.13	88.44	6.60	3.77
-	√	-	84.88	83.67	82.81	87.38	5.60	2.37
-	-	√	83.90	84.74	82.92	88.99	5.80	2.51
√	-	√	84.96	82.90	82.31	87.46	6.60	3.76
-	√	√	83.80	85.56	82.86	88.79	5.80	2.52
√	√	-	86.04	82.96	83.05	89.01	6.60	3.77
√	√	√	86.59	84.76	84.09	89.27	6.60	3.77

Note: The units of all metrics are the same as those in Table 4. “√” indicates the inclusion of the corresponding module, whereas “-” indicates its absence.

**Table 6:** Comparative experimental results of different segmentation networks.

Networks	mIoU/%	Dice/%	PA/%	FLOPs/G	Params/M
DeepLabV3	81.30	74.30	93.90	152.74	41.99
FCN	81.20	71.80	94.10	130.55	35.31
U^2^-Net	81.50	79.80	94.50	132.37	44.0
U-Net	82.40	76.60	94.60	141.29	17.26
VSG-UNet	83.90	81.80	95.00	78.81	6.70

**Table 7:** Ablation experimental results of VSG-UNet.

Module			mIoU	Dice	PA	FLOPs	Params
SG-Block	VGCA	GFF					
-	-	-	82.4	76.6	94.6	141.29	17.26
√	-	-	82.7	77.8	94.7	78.75	6.39
-	-	√	83.1	78.7	94.9	109.47	13.39
√	-	√	83.7	81.2	94.9	78.81	6.39
√	√	-	82.9	80.9	94.6	78.75	6.70
√	√	√	83.9	81.8	95.0	78.81	6.70

Note: The units of all metrics are the same as those in Table 6. “√” indicates the inclusion of the corresponding module, whereas “-” indicates its absence.

## Data Availability

The data supporting the findings of this study are not publicly available due to their classification as confidential.

## Abbreviations

The following abbreviations are used in this manuscript:

VDM-YOLO	Visual Dynamic Momentum You Only Look Once
VSG-UNet	Variance-Guided Strip-Ghost UNet
VGSG-Block	Variance-Guided Strip-Ghost Block
SG-Block	Strip-Ghost Block
VGCA	Variance-Guided Channel Attention
GFF	Gated Feature Fusion
ROI	Region of Interest
DySample	Dynamic Sampling Upsampling Module
C3K2_VSS	Visual State Space C3k2 Module
SS2D	Two-Dimensional State-Space Unit
Bottleneck_VSS	Visual State Space Bottleneck Module
mAP	Mean Average Precision
mAP_0.5_	Mean average precision at an intersection over union (IoU) threshold of 0.5
IoU	Intersection over Union
mIoU	Mean Intersection over Union
PA	Pixel Accuracy
*R*^2^	Coefficient of Determination
RMSE	Root Mean Square Error
CI	Confidence Intervals
MAE	Mean Absolute Error
MLP	Multi-Layer Perceptron
ReLU	Rectified Linear Unit
BN	Batch Normalization
FLOPs	Floating Point Operations
